# Understanding the relationship between body image and emotional distress among men and women with cancer

**DOI:** 10.1007/s00520-025-09703-3

**Published:** 2025-07-01

**Authors:** Janet Mokhnatkin, Marianne Razavi, Karen Clark, Ashley N. Hudson, Matthew Loscalzo, Jeanelle Folbrecht

**Affiliations:** 1 Department of Supportive Care Medicine, City of Hope National Medical Center, 1500 E. Duarte Rd, Duarte, CA 91010-3000 USA; 2 Department of Psychiatry and Behavioral Sciences, University of California, Davis Medical Center Behavioral Health Center, 2230 Stockton Blvd, Sacramento, CA 95817 USA

**Keywords:** Cancer, Body image, Physical appearance, Body function, Distress, Gender, Anxiety

## Abstract

**Purpose:**

This study addresses key gaps in knowledge regarding two components of body image that impact the emotional well-being of cancer patients: physical appearance and body function. The study examines the interrelationship of anxiety and gender in endorsement of distress related to body function and physical appearance.

**Methods:**

A retrospective study was conducted involving 1563 patients treated for cancer at City of Hope between 2009 and 2017. Patients completed a biopsychosocial problem-related distress touch-screen instrument before treatment as part of their routine clinical care. Relationships between body image constructs (physical appearance, body function), anxiety, and gender were examined using mixed models. Models controlled demographic (age, marital status, household income, education, and survey language) and clinical variables (cancer type, disease stage).

**Results:**

An association between anxiety and gender (*p* < 0.001) was found and was associated with body image distress. With higher levels of anxiety, women were significantly more distressed than men regarding physical appearance (*F* = 6.94, *p* = 0.009), while men were significantly more distressed than women regarding body function (*F* = 4.05, *p* = 0.044).

**Conclusion:**

Distress regarding body image is an important factor to consider in patients with cancer. Understanding the impact of anxiety and gender upon physical appearance distress and body function distress can inform individualized medical and psychosocial care.

## Background

Diagnosis and treatment of cancer has a profound emotional, physical, and functional impact. Cancer impacts not just the composition and make-up of one’s cells, but also the observable characteristics and function of one’s body. Research on body image distress has found that poor body image is most prevalent following surgery or during treatment [1] but continues into survivorship [2–4]. Body image distress is associated with poor self-esteem, depression [5–7], anxiety [5, 6], difficulty coping, and overall decrease in quality of life [8, 9]. Research suggests risk factors for body image distress include preexisting anxiety [8, 10] and female gender [4, 5, 9].

Body image distress is common across various cancer types, including breast, melanoma, head and neck, cervical, ovarian, colorectal, prostate, and blood cancers [6]. Approximately one-third of breast cancer patients [2], 68% of head and neck cancer (HNC) patients [10], and 36% of all cancer patients [5] endorse distress related to body image.

Body image has been conceptualized in numerous ways in literature. Most often, body image is considered a single construct. However, there is evidence to suggest the conceptualization of body image in two distinct constructs: physical appearance and body function [10–14]. Physical appearance encompasses feelings about changes in how the body looks and very frequently includes skin discoloration, hair loss, surgery scars, amputation, loss of hearing or sight, and weight gain or loss. For some oncology patients, surgical treatment or disease complications will lead to physical abnormalities. Invasive surgeries like mastectomy for breast cancer, limb loss in the context of osteosarcoma, testicular shrinkage or penile shortening for prostate patients, or facial disfigurement of head and neck cancer patients can dramatically affect one’s physical appearance.

Body function is an aspect of body image that encompasses changes in how the body performs or operates, including the ability to walk, eat, chew, swallow, participate in sexual activities, control urine or stool, provide self-care, or have children. Body function impacts one’s ability to live independently, return to work, participate in social/recreational activities, and enjoy their body. A comprehensive understanding of body image includes not only feelings about physical appearance but also how a patient feels about changes to how the body functions.

Most research of body image focuses exclusively on the construct of appearance and neglects the impact of function on body image distress. However, research on body image in breast cancer and head and neck cancer patients often uses physical appearance and body function constructs. Paterson researched body image and its impact on distress in breast cancer patients and found that poorer body image was related to both physical and psychological distress [3]. Rhoten explored the concepts of body image distress using similar constructs of *disfigurement* and *dysfunction* in head and neck cancer patients [7, 13]. Fingeret specifically compared distress related to physical appearance and body function and found patients with speech and eating concerns (i.e., body function concerns) had higher body image dissatisfaction than patients who only experienced appearance-based concerns. Additionally, patients with functional concerns were more likely to avoid social activities, perhaps contributing to poorer quality of life outcomes [1].

Few studies have examined differences between men and women oncology patients in the context of body image concerns. Even fewer have looked at gender differences on the physical appearance versus body function constructs of body image. The studies that have been conducted suggest that women might experience greater emotional distress related to physical appearance than men. In a study of adolescent and young adult cancer survivors, female patients reported more worries than male patients regarding physical appearance [4]. Similar results were found for women in active treatment and head and neck cancer patients [5, 9].

The literature suggests that men might experience greater emotional distress related to body function than women. A study conducted with young adult oncology patients revealed that male patients reported experiencing significantly greater distress related to eating, chewing, or swallowing and walking and climbing stairs than women [15]. Chopra found younger women were at higher risk of distress related to appearance but did not study concerns related to body function [5]. There is therefore reason to suggest body function might be of greater concern in men while physical appearance might be of greater concern for women.

An important variable to consider when exploring the construct of body image is anxiety. Anxiety is considered risk factor for cancer-related distress in general and body image specifically [8, 11]. Brederecke found that anxiety was a risk factor for body image dissatisfaction in women specifically rather than men [8]. Henry found body image distress at time of diagnosis was related to prior history of anxiety, while post-treatment body image concerns were related to “neuroticism,” among other factors [11]. Therefore, controlling the effects of overall anxiety is important when exploring body image and specifically the impact of gender on body image concerns.

The main aim of this study was to evaluate the interaction between anxiety and gender on the physical appearance and body function constructs of body image. It was hypothesized that with higher anxiety, women would report greater distress regarding physical appearance, while men would report greater distress regarding body function.

## Methods

### Participants and measures

This is a cross-sectional study (a secondary analysis of existing data) which included 1563 eligible patients with complete information regarding diagnosis, distress, and demographic characteristics. Demographic variables measured included age, marital status, household income, education, survey language, cancer stage, cancer type, and gender. This study was a retroactive study of data already collected, and the data was unfortunately limited in measuring gender as a binary variable.

Distress screening data was collected between 2009 and 2017 within a single NCI-designated Comprehensive Cancer Center. Patients had a waiver consent and were screened prior to treatment for their cancer. They used *SupportScreen®*, a touch screen-based mobile screening instrument, to report their problem-related distress. *SupportScreen* was offered in most but not all clinics, and the completion of questionnaires was voluntary. Each of the 30 distress items was scored on a 5-point Likert scale in which the participant rated how much a particular item was a problem, from 1 (not a problem) to 5 (very severe problem). This study was conducted using the ethical principles set forth in the World medical Association Declaration of Helsinki (1964), last updated in 2013. It was approved by the City of Hope National Medical Center Institutional Review Board (#08200).

Anxiety was measured using the *SupportScreen* item: feeling anxious or fearful. Distress related to physical appearance was measured using the *SupportScreen* single item: physical appearance. Distress related to body function was composed from a composite score of the following six problem-related distress items: walking or climbing stairs; eating, chewing, or swallowing difficulties; bowel movement or constipation; managing work, school, or home life; sleeping; fatigue. Sexual function was purposely excluded in order to focus on function related to daily activities. Although no factor analysis was conducted, this variable’s internal consistency was evaluated via Cronbach α (0.74) and had a potential range (6–30). Covariates studied included age, gender (male, female), marital status (married or partner versus not married, no partner), household income (< $40,000, $40,000–$100,000, $100,000 +), survey language (English and Spanish), education (< = HS, > HS), disease stage (early versus late), and cancer types.

### Statistical analysis

Descriptive statistics were generated for patients’ sociodemographic, clinical, and psychological characteristics and are presented as means (M, SD) or frequencies (*n*, %). Differences were tested via *t*-test or chi-square test. Distress means regarding physical appearance and body function were presented by site of cancer diagnosis. Distress means were also displayed by gender and cancer type (pairwise Bonferroni correction was applied).

The strength of the relationships between body image constructs and anxiety was analyzed using Pearson correlations. The effects of anxiety and gender (independent variables) on physical appearance and body function distress (dependent variables) were examined via general linear models (GLM). A separate model was produced for each dependent variable. The multivariable models included an interaction term (gender × anxiety) and were adjusted for the effects of covariates: age (continuous), marital status (married/partner, not married/no partner), household income (< $40,000, $40,000–$100,000, > $100,000), education (≤ HS, > HS), survey language (English and Spanish), disease stage (early and late), and cancer types (breast, digestive system, head and neck, gynecological, hematological, prostate, and urinary system).

For all the above analyses, statistical significance was assumed at *p* < 0.05. The models’ underlying assumptions were checked and met.

## Results

A total of 1563 patients were analyzed (complete cases). Patient characteristics are tabulated and presented in Table 1. The mean age was 60 (with men slightly older than women), and patients were mostly women (65%), English-speaking (96%), college-educated (70%), with a household income of less than $100,000 (77%). Men had more advanced disease stage (61%) than women (46%). The primary cancer types in this cohort consisted of breast (29%), gastrointestinal (18.5%), gynecological (14%), and lung (13%). The mean scores for anxiety, physical appearance, and body function were *M* = 1.99 (SD = 0.98, range 1–5), *M* = 1.57 (SD = 0.88, range 1–5) and *M* = 11.14 (SD = 4.08, range 6–30), respectively. The mean score for anxiety was higher for women than men (*M* = 2.11 vs *M* = 1.79, *p* < 0.001). Before controlling for the effect of anxiety, women reported higher physical appearance distress than men, and no difference between men and women was observed on body function.

**Table 1:** Descriptive statistics for demographic and study variables

	Total sample	Men	Women	*P*
	*N* = 1563	*N* = 550	*N* = 1013	
Demographic variables				
Age [*M* (SD)]	59.65 (13.14)	63.4 (12.6)	57.62 (12.99)	< 0.001
Marital status [*n* (%)]				< 0.001
Not married no partner	561 (35.9)	147 (25.7)	414 (40.9)	
Married or partner	1002 (64.1)	403 (73.3)	599 (59.1)	
Household income [*n* (%)]				< 0.001
$0–40,000	685 (43.8)	197 (35.8)	488 (48.2)	
$40,000–100,000	516 (33.0)	206 (37.5)	310 (30.6)	
$100,000 and over	362 (23.2)	147 (26.7)	327 (32.3)	
Education [*n* (%)]				0.003
< = HS	465 (29.8)	138 (25.1)	327 (32.3)	
> HS	1098 (70.2)	412 (74.9)	686 (67.7)	
Survey language [*n* (%)]				0.231
English	1496 (95.7)	531 (96.5)	965 (95.3)	
Spanish	67 (04.3)	19 (03.5)	48 (04.7)	
Cancer stage [*n* (%)]				< 0.001
Advanced	795 (50.9)	334 (60.7)	461 (45.5)	
Early	768 (49.1)	216 (39.3)	554 (54.5)	
Cancer type [*n* (%)]				< 0.001
Breast	455 (29.1)		455 (44.9)	
Digestive	289 (18.5)	146 (26.5)	143 (14.1)	
Head and neck	72 (04.6)	49 (08.9)	23 (02.3)	
Gynecological	225 (14.4)		225 (22.2)	
Hematological	88 (05.6)	48 (08.7)	40 (03.9)	
Prostate	168 (10.7)	168 (10.7)		
Lung	208 (13.3)	93 (16.9)	115 (11.4)	
Urinary	58 (03.7)	46 (08.4)	12 (01.2)	
Study variables [*M* (SD)]				
Feeling anxious/fearful	1.99 (00.98)	1.79 (00.86)	2.11 (01.03)	< 0.001
Body function	11.14 (04.08)	10.87 (04.22)	11.28 (04.00)	0.060
Physical appearance	1.57 (00.88)	1.37 (00.73)	1.67 (00.93)	< 0.001

Moderate correlations were observed between distress variables: *r* = 0.34 (body function and physical appearance); *r* = 0.45 (anxiety and body function); *r* = 0.43 (anxiety and physical appearance). All correlations were significant at *p* < 0.001.

Table 2 shows the average distress scores by cancer type. Of note, several cancer types had small sample sizes relative to the others, such as head and neck cancer, hematological cancers, and urinary cancers. Physical appearance distress was the highest for breast cancer patients (*M* = 1.75, SD = 1.02). Body function distress was the highest for patients with digestive system and lung cancers (*M* = 12.18 SD = 4.45, *M* = 12.16 SD = 4.45). Table 3 shows distress by gender and cancer types. For men, physical appearance distress was significantly higher for the head and neck cancer (*M* = 1.63) compared to prostate (*M* = 1.28), *p* = 0.043. Furthermore, significant differences were observed in body function distress between the digestive system and prostate cancers (*M* = 11.09 vs *M* = 9.53), *p* < 0.001, as well as between the lung and prostate cancers (*M* = 11.94 vs *M* = 9.53), *p* < 0.001. In women, body function distress differed significantly across several cancer types: breast (*M* = 10.76) versus digestive system (*M* = 12.48), *p* < 0.001; breast (*M* = 10.76) versus lung (*M* = 12.34), *p* = 0.003; and digestive system (*M* = 12.48) versus gynecological cancer (*M* = 11.05), *p* = 0.017.

**Table 2:** Distress by cancer type

Cancer type	*N*	Mean	SD
a) Physical appearance distress (range 1–5)			
Breast	455	1.75	1.02
Digestive system	289	1.48	0.80
Gynecological	225	1.60	0.77
Head and neck	72	1.68	1.05
Hematological	88	1.64	1.01
Lung	208	1.46	0.79
Prostate	168	1.28	0.60
Urinary	58	1.33	0.63
All patients	1563	1.57	0.88
b) Body function distress (range 6–30). Body function variable was computed using a composite score of 6 items			
Breast	455	10.76	3.634
Digestive system	289	12.18	4.452
Gynecological	225	11.05	4.137
Head and neck	72	10.75	4.107
Hematological	88	10.91	3.941
Lung	208	12.16	4.446
Prostate	168	9.53	3.412
Urinary	58	11.07	3.778
All patients	1563	11.14	4.079

Body function included 6 items, with each item rated on a 5-point Likert scale (ranging from 1 (not a problem) to 5 (severe problem)), leading to a total score range from 6 to 30 for the body function domain

**Table 3:** Distress by gender and cancer types

		Total	Breast	Digestive system	Head and neck	GYN	HEM	Prostate	Lung	Urinary
A. Physical appearance										
Women	*N*	1013	455	143	23	225	40	-	115	12
	Mean	1.67	1.75	1.58	1.78	1.6	1.88	-	1.55	1.5
Men	*N*	550	-	146	49	-	48	168	93	46
	Mean	1.37	-	1.39	1.63	-	1.44	1.28	1.35	1.28
B. Body function										
Women	*N*	1013	455	143	23	225	40	-	115	12
	Mean	11.28	10.76	12.48	11.39	11.05	11.23	-	12.34	11.08
Men	*N*	550	-	146	49	-	48	168	93	46
	Mean	10.87	-	11.9	10.45	-	10.65	9.53	11.94	11.07
C. Feeling anxious or fearful										
Women	*N*	1013	455	143	23	225	40	-	115	12
	Mean	2.11	2.1	2.05	2.39	2.13	1.88		2.2	1.83
Men	*N*	550	-	146	49	-	48	168	93	46
	Mean	1.79	-	1.95	1.88		1.77	1.68	1.81	1.57

Table A. Women: None of the pairwise comparisons (Bonferroni correction) were significantly different. Men: Pairwise comparisons (Bonferroni correction) showed significant differences regarding: head and neck and prostate (*p* = 0.043)

Table B. Women: None of the pairwise comparisons (Bonferroni correction) were significantly different. Men: Pairwise comparisons (Bonferroni correction) showed significant differences regarding: head and neck and prostate (*p* = 0.043)

Table C. Women: None of the pairwise comparisons (Bonferroni correction) were significantly different. Men: None of the pairwise comparisons (Bonferroni correction) were significantly different

**Fig. 1:**
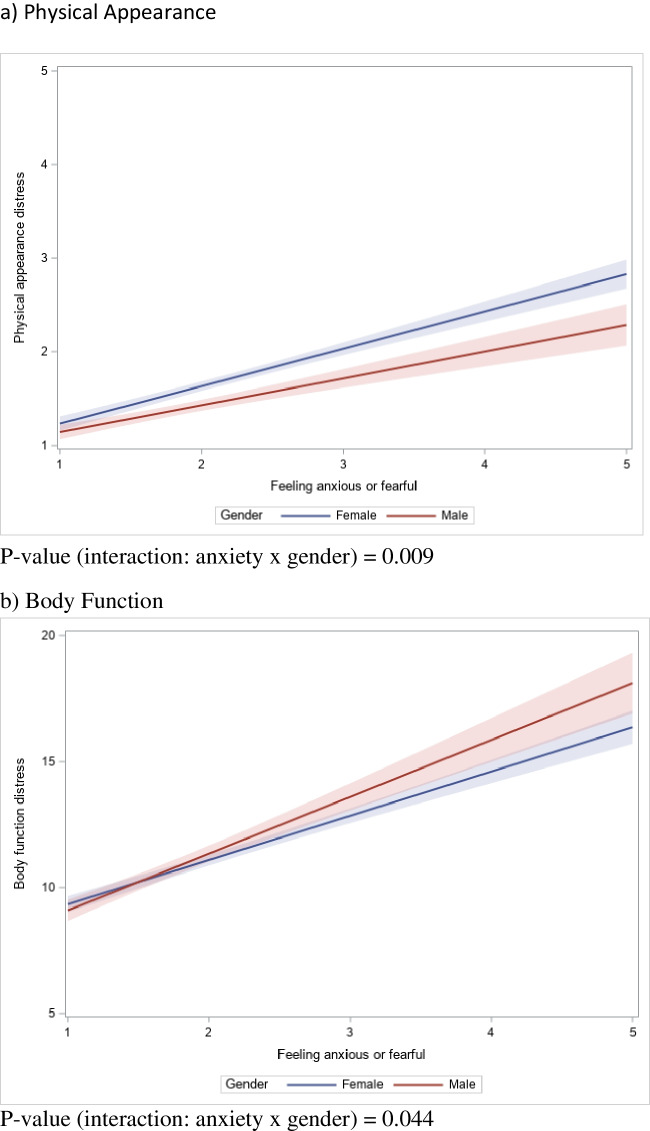
Relationship between body image distress (physical appearance and body function), anxiety, and gender: linear regression plots with 95% confidence limits

The GLM analyses (Table 4) showed a significant main effect of anxiety on physical appearance (*F* = 197.27, *p* < 0.001) and body function (*F* = 328.75, *p* < 0.001) for all patients. Notably, the results revealed a significant interaction effect between gender and anxiety on physical appearance and body function distress. These results (including gender differences) are visualized in Fig. 1. When there is no to minimal anxiety, women report more distress than men regarding physical appearance (*M* = 1.67 vs *M* = 1.37, *p* < 0.001), and there is no significant difference between women and men in distress regarding body function (*M* = 11.28 vs *M* = 10.87, *p* = 0.060). However, Fig. 1a shows that as anxiety increases, women report higher distress than men in physical appearance (*F* = 6.94, *p* = 0.009). Figure 1b suggests that as anxiety increases, men report higher distress in body function than women (*F* = 4.05, *p* = 0.044).

**Table 4:** Association between body image distress (physical appearance and body function), anxiety, and interaction gender × anxiety, adjusted to covariates

Variables	*F*-value	*P*-value
a) Physical appearance		
Gender	1.17	0.279
Feeling anxious or fearful	197.27	< 0.001
Feeling anxious or fearful × gender	6.94	0.009
Age	13.35	< 0.001
Marital status	0.93	0.336
Household income	1.83	0.161
Education	2.54	0.111
Survey language	5.79	0.016
Disease stage	0.40	0.527
Cancer type	1.79	0.085
b) Body function		
Gender	2.78	0.095
Feeling anxious or fearful	328.75	< 0.001
Feeling anxious or fearful × gender	4.05	0.044
Age	0.13	0.724
Marital status	1.43	0.233
Household income	22.04	< 0.001
Education	0.33	0.563
Survey language	1.79	0.181
Disease stage	17.68	< 0.001
Cancer type	5.18	< 0.001

Regarding physical appearance, GLM analyses (Table 4) showed significant main effects of age and survey language, with older patients reporting less distress (*F* = 13.35, *p* < 0.001), and patients completing the survey in Spanish (vs English) expressing more distress (*F* = 5.79, *p* = 0.016). For body function, lower household incomes were related to more distress (*F* = 22.04, *p* < 0.001). Furthermore, early disease stage was related to less body function distress (*F* = 17.68, *p* < 0.001).

## Discussion

### Principle findings

This paper explored the impact of gender and anxiety on the body image distress constructs of physical appearance and body function, constructs previously suggested in the breast cancer and head and neck body image literature [1, 10, 12, 13]. We used items from *SupportScreen* to measure anxiety and distress related to physical appearance and body function in patients with multiple cancer diagnoses. Specifically, the biopsychosocial screening tool implemented and used at City of Hope is a valid instrument for identifying psychological distress, as demonstrated in Wong et al. (2019) [18]. The moderate correlation between body image variables supports the idea that physical appearance and body function are two related but distinct constructs.

Our results demonstrated an interaction between gender and anxiety such that as anxiety increases, women were significantly more concerned than men about physical appearance, and men were significantly more concerned than women about body function. Physical appearance distress was found to be highest in breast cancer patients, while body function distress was found to be highest in digestive system cancer patients.

Although caution must be used when examining distress by cancer type due to small sample size in cancer types such as head and neck cancer, several interesting differences were found and should be further explored in future studies. For men, physical appearance distress was significantly higher in head and neck cancer patients compared to prostate cancer patients. Body function distress was found to be significantly higher in men with digestive system cancer as opposed to men with prostate cancer; it was also found to be significantly higher in men with lung cancer as opposed to men with prostate cancer. Conversely, there were no significant differences found in women with different cancer types with regards to physical appearance distress. Women with breast cancer reported significantly lower body function distress compared to women with digestive system cancer or lung cancer, and women with digestive system cancer were found to have significantly more body function distress than women with gynecological cancer.

Additional risk factors for distress regarding physical appearance were younger age and taking the survey in Spanish. Additional risk factors for distress regarding body function were lower household income, higher disease stage, and cancer type.

#### Clinical implications

The implications of these findings for body image interventions are twofold. First, body image overall is an important part of cancer treatment and should be included in assessments of distress and determination of need for intervention. Second, research on interventions for physical appearance and body function distress separately is needed. Literature has explored and evaluated interventions for body image distress even if not examining interventions for physical appearance or body function separately. A recent review of the interventions for body image distress examined eight intervention studies and classified them into four types: 1) psychotherapy groups, 2) peer group, 3) physical or exercise interventions, and 4) cosmetic/beauty interventions [16]. Of the four, psychotherapy and peer groups were found to be most effective. Online psychotherapy groups, self-guided interventions, and individual tele-therapy programs demonstrate promise as well [17]. Furthermore, the influence of media, societal standards, and cultural norms on body image distress, and the difference in prevalence and impact between men and women, must be taken into consideration, as well as be factored into psychotherapy and other targeted interventions.

Although psychotherapy and peer groups are important in managing body image distress, attention to appearance and physical interventions remains important when considering gender differences. Cognitive-behavioral therapy groups can be effective in addressing beliefs and attitudes towards appearance that lead to avoidance or poor self-evaluation. Also, Positive Image Centers are an intervention for physical appearance by providing support in the realm of cancer’s visible side effects. Services range from complimentary haircuts and head shaves to custom wig fittings and cuts. Because women demonstrate more distress than men regarding physical appearance at any level of anxiety, the interventions are usually geared towards the appearance of female patients, including post-mastectomy bra and camisole fittings, breast prostheses, lymphedema sleeves, and makeup application. Furthermore, it would be pertinent to explore the needs of patients in long-term survivorship in contrast with their needs immediately after diagnosis—how these needs change over time could help inform intervention strategies.

Interventions for body function concerns are provided through rehabilitation and include physical therapy, occupational therapy, and speech therapy. Men or women experiencing challenges with sexual dysfunction, often resulting from medical treatments for cancer, should be made aware of rehabilitation programs to improve function. Future exploration of treatment outcomes specific to physical appearance and body function could help refine clinical treatment pathways for body image distress.

### Limitations

There are several limitations to this study. One limitation is that the research was an observational study, conducted early in diagnosis, and that there is no data on changes in distress and physical appearance or body function throughout and following treatment. This study relies on cross-sectional data, which restricts the ability to draw conclusions about causality and the progression of distress over time. Future research should involve longitudinal assessment of body image concerns and changes in those concerns throughout treatment, the differences between men and women, and the differences in cancer type and treatment modalities. This would provide information on the impact of type of treatment, type of cancer, and gender on timing of interventions for the care provider.

It is also significant to note that distress scores were low overall (below 2.5 for physical appearance distress, as well as below 15 for body function distress); this could be due to a variety of reasons, including the limitation of being a study conducted early in diagnosis. This finding bolsters further need for future research involving changes in physical appearance distress and body function distress over time.

An additional limitation is the reliance on self-report measures of distress, including overall anxiety (susceptible to rater bias), physical appearance distress, and body function distress. Also, it may be pertinent to utilize other screening tools to further draw conclusions about the level of anxiety experienced. The use of one item for body image may lead to inaccurate conclusions. Future research could include validated multi-item scales specifically developed to measure anxiety (such as GAD-7). Also, including mixed method design (e.g., qualitative interviews) would provide telling information on patient perspectives, attitudes, and narratives.

Another limitation is that *SupportScreen* did not address aspects of intimate life and sexuality, which are important factors that may play a significant role in patients’ quality of life. Therefore, the results of analyses that include body function might underestimate the role of overall body dysfunction on distress. Future measures of body function should include sexual dysfunction so that the impact on patient distress can be further understood, contributing to the effectiveness of interventions.

### Future research

Data was collected in the years preceding the increased awareness in gender diversity that came during the COVID-19 pandemic. Future research should include data that is more gender inclusive by collecting information on sex assigned at birth and gender identity rather than a “male” or “female” forced choice. Including transgender and non-binary individuals would enhance study inclusivity and understanding of the relationship between gender and body image. Furthermore, including sexual function as part of body function distress and conducting the research longitudinally would shed additional light on patient body image distress and work towards assisting in development of effective interventions.

## Conclusion

Body image concerns, including distress regarding physical appearance and distress regarding body image, are prevalent among patients with cancer. This study found the interaction of gender and anxiety to be an important predictor of distress regarding physical appearance and body function. The significance lies in highlighting the need to address risk factors for both in clinical and research settings. Furthermore, clinical recommendations for addressing this distress include psychotherapy, peer groups, cognitive-behavioral therapy, and utilization of resources and expertise as found in the Positive Image Center. This work will further pave the way to improving the cancer patient’s journey through treatment and into survivorship.

## Data Availability

No datasets were generated or analysed during the current study.

## Materials availability

The data that support the findings of this study are available from the corresponding author upon reasonable request.

## Code availability

N/A.
